# Mechanisms of chronic pain

**DOI:** 10.1186/1744-8069-10-S1-O15

**Published:** 2014-12-15

**Authors:** Joachim Scholz

**Affiliations:** 1 Departments of Anesthesiology and Pharmacology, Columbia University Medical Center, New York, NY 11201, USA

## 

Chronic pain is classified as nociceptive or neuropathic, depending on whether the integrity of the somatosensory nervous system is compromised by the underlying disease. Nociceptive pain results from the activation of receptors (nociceptors) sensitive to noxious stimuli. Prolonged or intense exposure to these stimuli, for example, chemical mediators released during inflammation, enhances the responsiveness of nociceptive nerve fibers [1]. This process, termed peripheral sensitization, involves a shift in the activation threshold of nociceptors and upregulation of voltage-gated sodium channels. Peripheral sensitization leads to increased action potential firing and transmitter release in the dorsal horn of the spinal cord, where somatosensory information is processed. Dorsal horn neurons react to the rising input with heightened excitability, a process termed central sensitization. Enhanced depolarization leads to the recruitment of N-methyl-D-aspartate (NMDA)-type glutamate receptors. NMDA and neuropeptide receptor activation produces a sharp increase in intracellular calcium, triggering signaling pathways and gene expression changes that promote a long-term shift in the activity of nociceptive circuits [2]. In some aspects, central sensitization even resembles long-term potentiation of excitatory transmission in the hippocampus. Central sensitization generates an exaggerated response to painful stimuli (hyperalgesia) and contributes to pain elicited by normally nonpainful stimuli (allodynia). Clinical findings suggest that pain hypersensitivity produces structural changes in the brain over time. These changes are, however, reversible upon pain relief. The pathophysiology of neuropathic pain is fundamentally different. Peripheral nerve lesion evokes stimulus-independent (ectopic) activity in nerve fibers. Innate immune cells react at the lesion site, in the dorsal root ganglion, where the cell bodies of peripheral somatosensory neurons reside, and in the dorsal horn of the spinal cord [3]. Active microglia of the dorsal horn releases chemical mediators that modulate the activity of neurons in the vicinity. One of these mediators, brain-derived neurotrophic factor, reduces the inhibitory effect of γ-aminobutyric acid (GABA) and glycine. Disinhibition opens polysynaptic connections in the dorsal horn, further enhancing the abnormal input from the lesioned nerve. Similarly to nociceptive pain, central sensitization occurs. Worsened by a relative deficit in transmitter uptake, increased glutamatergic transmission causes excitotoxic cell death, reducing the number of inhibitory interneurons. Their loss and a shift in descending modulatory pathways from the brainstem produce a profound imbalance between inhibition and excitation. The complexity of chronic pain mechanisms poses a major therapeutic challenge. Without biomarkers, it will remain difficult to develop targeted strategies for chronic pain reduction or prevention in the individual patient.

## Disclosures

No conflict of interest.
